# Isolation and immortalization of macrophages derived from fetal porcine small intestine and their susceptibility to porcine viral pathogen infections

**DOI:** 10.3389/fvets.2022.919077

**Published:** 2022-07-18

**Authors:** Takato Takenouchi, Kentaro Masujin, Ayako Miyazaki, Shunichi Suzuki, Michihiro Takagi, Takehiro Kokuho, Hirohide Uenishi

**Affiliations:** ^1^Institute of Agrobiological Sciences, National Agriculture and Food Research Organization, Tsukuba, Japan; ^2^Division of Transboundary Animal Disease Research, National Institute of Animal Health, National Agriculture and Food Research Organization, Kodaira, Japan; ^3^Division of Infectious Animal Disease Research, National Institute of Animal Health, National Agriculture and Food Research Organization, Tsukuba, Japan

**Keywords:** African swine fever virus, immortalized porcine intestinal macrophages, *in vitro* model, porcine reproductive and respiratory syndrome virus, porcine small intestine macrophages

## Abstract

Macrophages are a heterogeneous population of cells that are present in all vertebrate tissues. They play a key role in the innate immune system, and thus, *in vitro* cultures of macrophages provide a valuable model for exploring their tissue-specific functions and interactions with pathogens. Porcine macrophage cultures are often used for the identification and characterization of porcine viral pathogens. Recently, we have developed a simple and efficient method for isolating primary macrophages from the kidneys and livers of swine. Here, we applied this protocol to fetal porcine intestinal tissues and demonstrated that porcine intestinal macrophages (PIM) can be isolated from mixed primary cultures of porcine small intestine-derived cells. Since the proliferative capacity of primary PIM is limited, we attempted to immortalize them by transferring the SV40 large T antigen and porcine telomerase reverse transcriptase genes using lentiviral vectors. Consequently, immortalized PIM (IPIM) were successfully generated and confirmed to retain various features of primary PIM. We further revealed that IPIM are susceptible to infection by the African swine fever virus and the porcine reproductive and respiratory syndrome virus and support their replication. These findings suggest that the IPIM cell line is a useful tool for developing *in vitro* models that mimic the intestinal mucosal microenvironments of swine, and for studying the interactions between porcine pathogens and host immune cells.

## Introduction

Macrophages are phagocytes that play essential roles in the innate immune system (1, 2). They contribute to the removal of harmful foreign particles, bacteria, and dying or dead cells. Macrophages are present in all of the tissues in the body and are involved in both the maintenance of tissue homeostasis and the progression of various inflammatory diseases (2). Previously, it was considered that they circulate in the blood as bone marrow (BM)-derived monocytes before entering tissues and differentiating into macrophages with tissue- and niche-specific functions (3). However, recent studies have revealed that most tissue-resident macrophages originate from the yolk sac and/or fetal liver during embryonic development (4), and circulating monocytes contribute minimally to tissue macrophages in steady state conditions (5).

Since the gastrointestinal (GI) tract faces unrivaled exposure to foreign antigens, it is the largest part of the body's immune system. Macrophages are recognized as essential players in the gut mucosal immune system, and intestinal macrophages constitute the largest pool of macrophages in the body (6). They are also implicated in the progression of chronic GI pathologies, such as inflammatory bowel disease (7). As for the origin of intestinal tissue-resident macrophages, recent studies have demonstrated that they are composed of embryo-derived macrophages prior to birth and are subsequently replaced by BM-derived monocytes with age (4, 8, 9).

Primary macrophage cultures are a valuable *in vitro* model for exploring the tissue-specific functions of macrophages and their interactions with pathogens. Previous studies have reported simple and efficient methods for isolating and propagating tissue-resident macrophages from various rodent organs (10–13). These protocols involve the following common steps: (1) the propagation of macrophages in a co-culture with stromal cells of the relevant organs/tissues using standard tissue culture-grade dishes (TC-dishes) or flasks, and (2) the isolation of macrophages on the basis of their adhesion to non-tissue culture-grade petri dishes (NTC-dishes).

Porcine macrophages have often been used for the identification and characterization of porcine viral pathogens. Regarding their primary culture, Talbot et al. reported that a cell sheet made of STO mouse fibroblast cells can be utilized as a feeder layer for expanding and culturing porcine tissue-resident macrophages from various organs (14, 15). Based on a protocol for isolating rodent macrophages, simple and efficient methods for isolating porcine macrophages were proposed in our previous studies (16, 17). These methods involved mixed primary cultures of porcine kidney or liver-derived cells, in which stromal cells from the relevant tissues served as feeder cells.

In the present study, we applied this protocol to fetal porcine intestinal tissues and demonstrated that porcine intestinal macrophages (PIM) can be isolated from a mixed primary culture of porcine small intestine-derived cells. In addition, we established a novel immortalized PIM (IPIM) cell line using a previously described porcine macrophage immortalization protocol (18) and demonstrated that the IPIM are susceptible to infection by the African swine fever virus (ASFV) and porcine reproductive and respiratory syndrome virus (PRRSV). It is expected that the IPIM cell line will be a useful tool for developing an *in vitro* model of transmissible gastroenteritis in pigs.

## Materials and methods

### Ethics statement

The protocols for the use of animals were approved by the animal care committee of the Institute of Agrobiological Sciences (#H28-P04) and the National Institute of Animal Health (NIAH) (#20-046), National Agriculture and Food Research Organization (NARO). Animal experiment procedures were carried out in accordance with the regulations outlined in Guide for the Care and Use of Laboratory Animals of the Institute of Agrobiological Sciences and the NIAH, NARO, Guidelines for Proper Conduct of Animal Experiments of the Science Council of Japan and the ARRIVE guidelines (19). The experiments involving lentiviral vectors were approved by the gene recombination experiment safety committee of the Institute of Agrobiological Sciences (#1036465) and the NIAH (#A-20-002 and # A-20-005), NARO.

### Isolation of PIM

The small intestine (the ileum, around 20 cm long) was dissected out from a crossbred male porcine fetus at 108 days of gestation and cut into small pieces with scissors, and the tissue pieces were digested by incubating them with collagenase-dispase (Roche Diagnostics, Basel, Switzerland)/Dulbecco's phosphate-buffered saline (DPBS) solution (1 mg/mL) containing DNase I (Roche Diagnostics) (40 μg/mL) for 1 h at 37°C. Then, the digested tissue fragments were collected and re-suspended in growth medium composed of Dulbecco's modified Eagle's medium (DMEM) (Sigma, St. Louis, MO) containing 10% heat-inactivated fetal bovine serum (FBS) (FUJIFILM Wako Pure Chemical Corp., Osaka, Japan), supplemented with 25 μM monothioglycerol (FUJIFILM Wako), 10 μg/mL insulin (Sigma), streptomycin-penicillin (100 μg/mL and 100 U/mL, respectively) (Nacalai Tesque, Inc., Kyoto, Japan), and 5 μg/mL Fungin (InvivoGen, San Diego, CA). The tissue suspension was added to T-75 tissue culture flasks (Sumitomo Bakelite Co., Ltd., Tokyo, Japan) with 10 mL growth medium and cultured at 37°C in a humidified atmosphere of 95% air/5% CO_2_ (CO_2_ incubator BNA-111, ESPEC CORP., Osaka, Japan). The culture medium was replaced every 3–4 days. After 2–3 weeks, the attached cells were harvested by treating them with Accumax^TM^ (Innovative Cell Technologies, Inc., San Diego, CA), and then the cell suspensions were re-plated in 100-mm TC-dishes (Cat. No. 353003, Corning Incorporated, Corning, NY). After 1–2 weeks, a sheet-like mixed cell monolayer formed, and macrophage-like cells began to proliferate on the cell sheet. The proliferating cells were loosely attached to the cell sheet and so were harvested from the culture supernatant by centrifugation (1,500 rpm for 5 min). Since macrophages readily attach to NTC-dishes (Sumitomo Bakelite Co., Ltd.), they were selectively isolated from the other types of cells on the basis of this feature (16, 17).

### Establishment of IPIM

Lentiviral particles carrying the SV40 large T antigen (SV40LT) gene and porcine telomerase reverse transcriptase (pTERT) gene were prepared as described previously (18). To generate lentiviruses, pLVSIN-EF1α neo vectors (Takara Bio, Inc, Shiga, Japan.) encoding SV40LT or pTERT were co-transfected with packaging vectors (lentiviral high titer packaging mix) (Takara Bio) into Lenti-X 293T cells (Takara Bio) using the TransIT-293 transfection reagent (Takara Bio) according to the manufacturer's instructions. PIM were transfected with these lentiviral particles in the presence of 6 μg/mL of Polybrene (Nacalai Tesque). The proliferating cells were cultured with growth medium containing 800 μg/mL G418 (Nacalai Tesque) to select resistant cells.

For the IPIM subculturing, cells (1 × 10^6^) were seeded in 90-mm NTC-dishes (Cat. No. MS-1390R, Sumitomo Bakelite Co., Ltd.) and continuously passaged every 5–6 days. At each passage, the cells were detached using TrypLE express solution (Thermo Fisher Scientific, Waltham, MA), and the number of harvested cells was measured using a Bio-Rad TC10 automated cell counter.

### Immunocytochemistry

Cells were seeded in 8-well-chamber slides (Asahi Glass Co., Ltd., Tokyo, Japan) at a density of 1.5~2 × 10^5^ cells/well. After being washed once with DPBS, the cells were fixed using 4% paraformaldehyde phosphate buffer solution (Nacalai Tesque) for 15 min at room temperature. After being washed with PBS containing 0.05% Tween 20 (PBST), the cells were permeabilized with 1% Triton X-100/PBS solution for 10 min and blocked with Blocking One Histo (Nacalai Tesque) for 30 min. Then, the cells were incubated with the primary antibodies diluted 1:400 with PBST (around 2.5 μg/mL) for 1 h at room temperature in a humidified box. After rinsing the slides with PBST, the EnVision system (DAKO, Hamburg, Germany) was used to visualize antibody-antigen reactions, according to the manufacturer's procedure. Cell nuclei were counterstained with Mayer's hematoxylin solution (FUJIFILM Wako). The stained slides were examined under a microscope (Leica, Bensheim, Germany).

The primary antibodies used in this study were as follows: mouse monoclonal antibodies against α-smooth muscle actin (αSMA) (Progen, Heidelberg, Germany), vimentin (Progen), CD172a (VMRD, Inc., Pullman, WA), cytokeratin 18 (CK18) (Millipore Co., Billerica, MA, USA), cytokeratin 19 (CK19) (Progen), macrophage scavenger receptor A (MSR-A: CD204; TransGenic, Inc., Kumamoto, Japan), CD203a (Bio-Rad, Hercules, CA), CD16 (Bio-Rad), CD163 (Bio-Rad), CD169 (Bio-Rad), and major histocompatibility complex class II (MHC-II) (Kingfisher Biotech, Inc., St. Paul, MN); and a rabbit polyclonal antibody against ionized calcium-binding adaptor molecule 1 (Iba1) (FUJIFILM Wako).

### Polymerase chain reaction-based sex determination

Porcine amelogenin (*AMEL*) genes, which are present on both the X- (*AMELX*) and Y-chromosomes (*AMELY*), were used for identifying the sex of the cells. In a previous study, primer pairs that yield different-sized PCR products for each sex were designed (20); i.e., genomic DNA from male pigs produces two bands (520 and 350 bp; *AMELX* and *AMELY*, respectively), whereas genomic DNA from female pigs generates only 520-bp products. IPIM or IPKM (2 × 10^4^) were directly added as templates for PCR amplification using KOD FX DNA polymerase (Toyobo Co., Ltd., Osaka, Japan), according to the manufacturer's instructions. The PCR reaction buffer (50 μL) contained 0.4 mM dNTPs, oligonucleotide primers (0.3 μM each) and 1.0 unit KOD FX. Fifty temperature cycles were conducted as follows: denaturation at 98°C for 10 s; annealing at 58°C for 30 s; and extension at 68°C for 30 s in TaKaRa PCR Thermal Cycler Dice (Takara Bio). The PCR products were analyzed by agarose gel electrophoresis and visualized *via* GelGreen^TM^ staining (Biotium, Inc., Fremont, CA).

### Immunoblotting

IPIM (4 × 10^5^ cells/well in a 24-well-plate) were stimulated with MDP (InvivoGen) or LPS (Sigma) in the serum-free DMEM at the concentrations indicated. After being incubated at 37 °C for 3 days, the culture supernatants were collected, and the cells were lysed with 200 μL ice-cold lysis buffer [50 mM Tris-HCl (pH 7.4), 150 mM NaCl, 0.5% Triton X-100, and 0.5% sodium deoxycholate] containing cOmplete^TM^ mini protease inhibitor (Roche). The production of interleukin-1α (IL-1α) and IL-1β were evaluated by immunoblotting, as described previously (18). In brief, equal volumes of culture supernatant and cell lysate (25 μL) were separated by sodium-dodecyl sulfate-polyacrylamide gel electrophoresis and electro-blotted onto a polyvinylidene difluoride membrane (Millipore). To detect IL-1α and IL-1β, the membranes were incubated with biotinylated anti-porcine IL-1α or IL-1β antibody (1:1,000) for 1 h; followed by incubation with horseradish peroxidase-conjugated streptavidin (1:5,000) for 20 min. The target protein was revealed using Chemi-Lumi One ultra (Nacalai Tesque) and detected using a C-DiGit blot scanner (LI-COR, Inc., Lincoln, NE).

### ASFV titration

The ASFV field isolates Armenia07, Kenya05/Tk-1, and Espana75 were courteously provided by Dr. Sanchez-Vizcaino (Universidad Complutense de Madrid, Spain). These isolates were routinely maintained in primary porcine alveolar macrophages (PAM) cell cultures and stored in aliquots at −80°C until use. PAM were prepared from 8-week old Landrace-Large White-Duroc crossbred pigs using broncho-alveolar lavage procedure as described previously (21), and cultured in RPMI1640 (Nacalai Tesque) supplemented with 10% FBS and antibiotics. The Lisbon60 isolate was kindly provided by Dr. Genovesi (Plum Island Animal Disease Center, USA) and serially passaged in Vero cell cultures to establish the Vero cell-adapted Lisbon60V viruses.

Virus titrations for the ASFV isolates were analyzed using cytopathic effects (CPE) and hemadsorption (HAD) assays, as described in a previous paper (22). Briefly, IPIM and PAM cells were seeded in 96-well-cell culture plates, and 100 microliters of 10-fold serially diluted samples were inoculated into the wells. The presence of CPE was examined by microscopy. HAD assays were performed using porcine red blood cells, and the presence of rosette formation was examined by microscopy. To evaluate ASFV production, IPIM and PAM cells were seeded in 24-well-cell culture plates, and inoculated with ASFV isolates (Armina07, Kenya05/Tk-1 and Lisbon60V) at a multiplicity of infection (MOI) of 0.01. After incubation for 1 h, the inoculum was removed, the cells were washed three times with DPBS, and then the growth medium was newly added. The culture supernatants were collected at 1, 2, 3, 4, and 5 days post-inoculation (dpi) and viral titers were examined in the IPIM cell cultures. All experiments with ASFV were performed at the Biosafety Level 3 facility of NIAH and were approved by the Japanese national authority (Permit No. 32).

### Statistical analysis

The Student's *t*-tests were applied on paired data to determine statistical significance. Differences with *p* < 0.05 were considered significant. Statistical analysis was performed using KaleidaGraph software (Synergy Software, Reading, PA, USA).

### Immunofluorescence assay

The first Japanese PRRSV field isolate, the EDRD-1 strain (23), was propagated in PAM and stored in aliquots at −80°C until use. IPIM were seeded in 96-well NTC-plates (Sumitomo Bakelite Co., Ltd.) at a density of 1 × 10^5^ cells/well and pre-cultured for 3 days in the growth medium at 37°C. Then, the cells were inoculated with the PRRSV EDRD-1 strain at a MOI of 0.01, before being incubated at 37°C for 3 days. After being washed once with DPBS, the cells were fixed with 80% acetone for 10 min on ice and incubated with a fluorescein isothiocyanate (FITC)-conjugated anti-PRRSV mouse monoclonal antibody SR30 (1: 50,000 in PBS) (RTI, Brookings, SD, USA) for 1 h at room temperature. Fluorescent signals were observed under a fluorescence microscope (Carl Zeiss, Jena, Germany).

### PRRSV titration

IPIM (3 × 10^6^ cells/well) were seeded in 6-well NTC-plates (Sumitomo Bakelite Co., Ltd.) and pre-cultured for 3 days. Then, the cells were inoculated with the PRRSV EDRD-1 strain at an MOI of 0.01, before being subjected to virus absorption at 37 °C for 1 h. After being washed twice with serum-free DMEM; 5 mL growth medium was newly added to the culture; and 0.5-mL culture supernatant samples were collected at 1, 2, 3, 5, and 7 dpi and stored at −80°C until use. One hundred microliters of 10-fold serially diluted supernatant samples were inoculated into the wells of the 96-well NTC-plates (in quadruplicate) in which IPIM (1 × 10^5^ cells/well) had been pre-cultured for 3 days in the growth medium at 37°C. After 7 days' culture, the presence/absence of CPE was examined by microscopy (CK2, OLYMPUS, Tokyo, Japan), and the titer of each sample was calculated using the Behrens-Karber method (24). The results were representative of three independent experiments and were expressed as the mean of three replicates.

## Results

### Isolation and characterization of PIM

**Figure 1:**
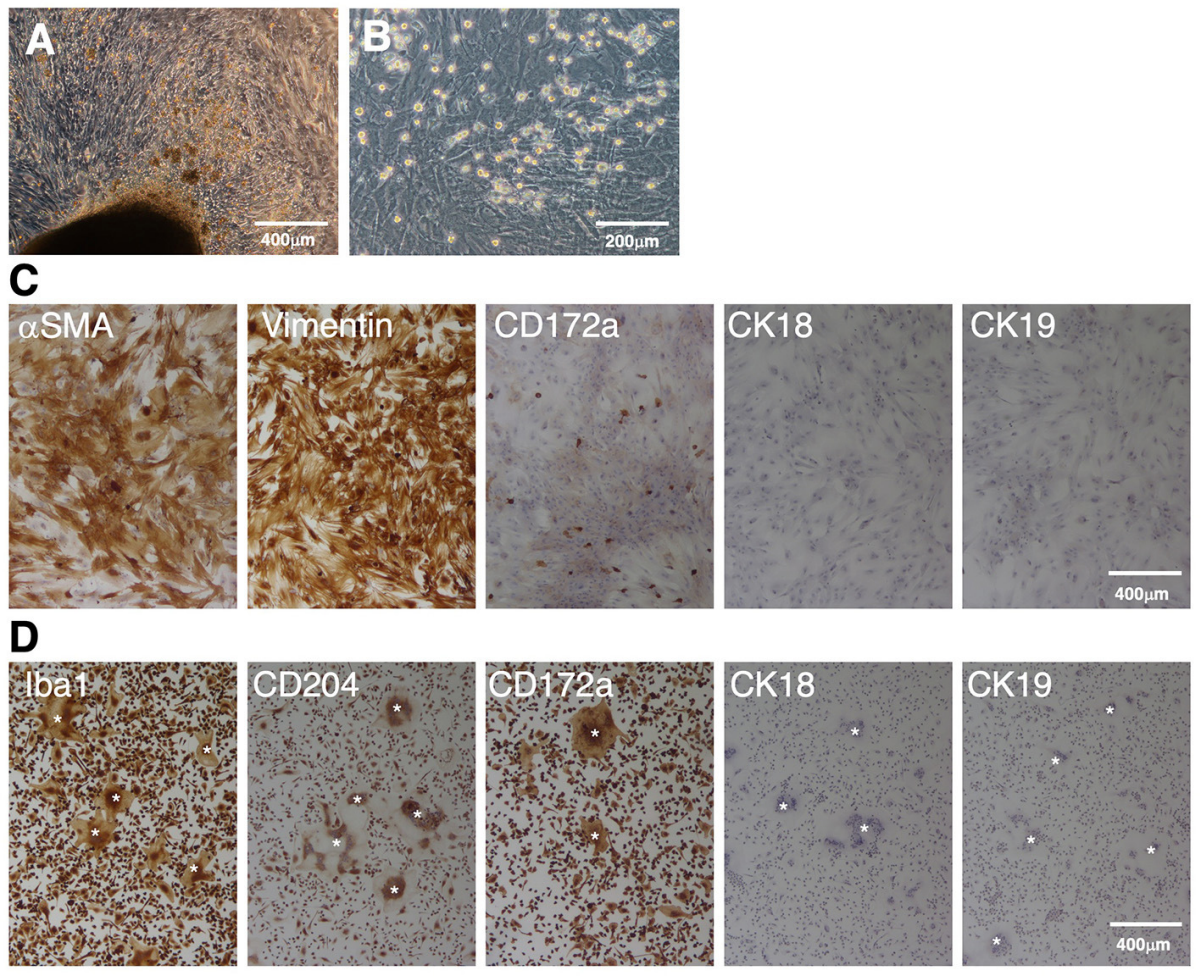
Immunocytochemical characterization of a mixed primary culture of fetal porcine small intestine-derived cells and isolated PIM. Primary intestinal cells cultured in T-75 tissue culture flasks **(A)** or TC-dishes **(B)** were observed under a phase-contrast microscope. The cells were seeded in 8-well-chamber slides, fixed using 4% paraformaldehyde phosphate buffer solution, and immunostained with specific antibodies against mesenchymal (αSMA and vimentin), macrophage (CD172a), or epithelial (CK18 and CK19) cell markers [**(C)**, brown]. The PIM isolated from the supernatant samples of the mixed primary culture were immunostained with specific antibodies against macrophage (Iba1, CD204, and CD172a) or epithelial (CK18 and CK19) cell markers [**(D)**, *brown*)]. Multinucleated giant cells were sometimes observed in the PIM culture [**(D)**, *white asterisks*]. All nuclei were counterstained with hematoxylin [**(C,D)**, *blue*]. Images are representative of two independent experiments.

When enzyme-digested tissue suspensions of fetal porcine small intestines were seeded in T-75 tissue culture flasks, the primary cells attached to the flask surface and expanded from the tissue fragments within 3 weeks (Figure 1A). Then, the cells were harvested using enzymatic treatment and passaged in 100-mm TC-dishes. After 1–2 further weeks of culture, a confluent cell sheet formed on the TC-dishes, and round cells that were loosely attached to the cell sheet were propagated (Figure 1B). Immunostaining showed that the sheet-forming cells were positive for mesenchymal cell markers (αSMA and vimentin), but negative for epithelial markers (CK18 and CK19) (Figure 1C). The round cells observed on the cell sheet were positive for the macrophage marker CD172a (Figure 1C).

The round cells were collected from the culture supernatant, and almost purely PIM were isolated from other types of cells based on their adhesion property to NTC-dishes. As shown in Figure 1D, almost all of the isolated cells were positive for macrophage markers (Iba1, CD204, and CD172a), but negative for epithelial cell markers (CK18 and CK19). The spontaneous formation of multinucleated giant cells from isolated PIM was sometimes observed, which is a feature of primary macrophages in culture (Figure 1D).

### Establishment and characterization of IPIM

**Figure 2:**
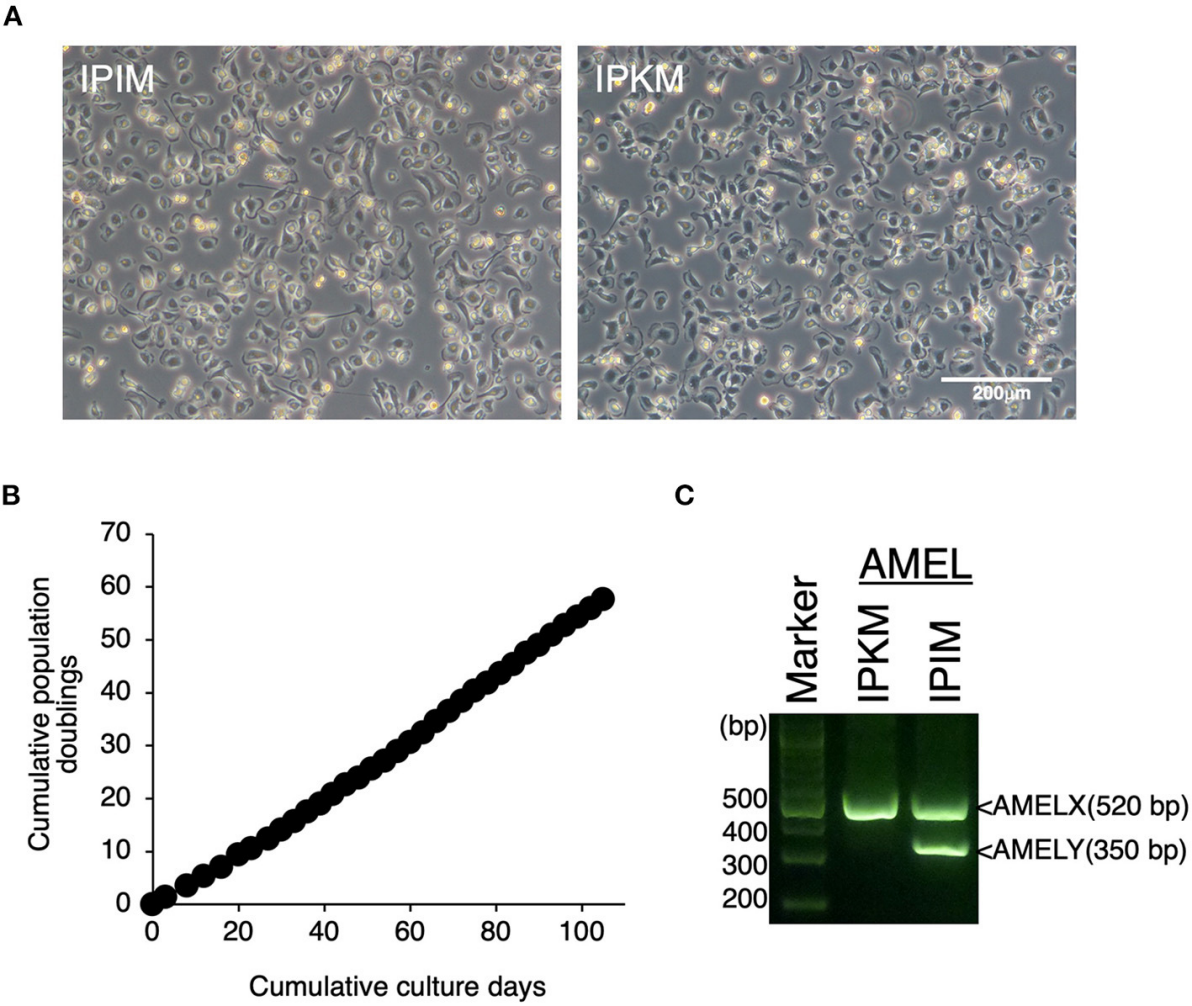
Establishment of IPIM and comparison of their features with IPKM. The morphologies of IPIM and IPKM were observed under a phase-contrast microscope **(A)**. The cumulative population doublings of the IPIM were plotted against the duration of the culture period (in days) **(B)**. PCR-based sex identification based on the porcine amelogenin (*AMEL*) genes was performed using genomic DNA from the IPIM and IPKM, and PCR products derived from *AMELX* (520 bp) and *AMELY* (350 bp) were analyzed by agarose gel electrophoresis **(C)**.

Since the proliferation of isolated PIM ceased under standard culture medium conditions, we attempted to immortalize these cells. Eventually, IPIM were successfully generated by transferring both the SV40LT and pTERT genes with lentiviral vectors. They exhibited a typical macrophage-like morphology with ruffled membranes and cell processes (Figure 2A, left). The IPIM proliferated at a doubling time of around 2 days and were stably passaged for >50 population doublings (Figure 2B). Their morphology resembled that of the immortalized porcine kidney-derived macrophages (IPKM) established previously (Figure 2A, right). However, PCR-based sex determination analysis showed that the IPIM originate from a male pig, while the IPKM originate from a female pig (Figure 2C).

**Figure 3:**
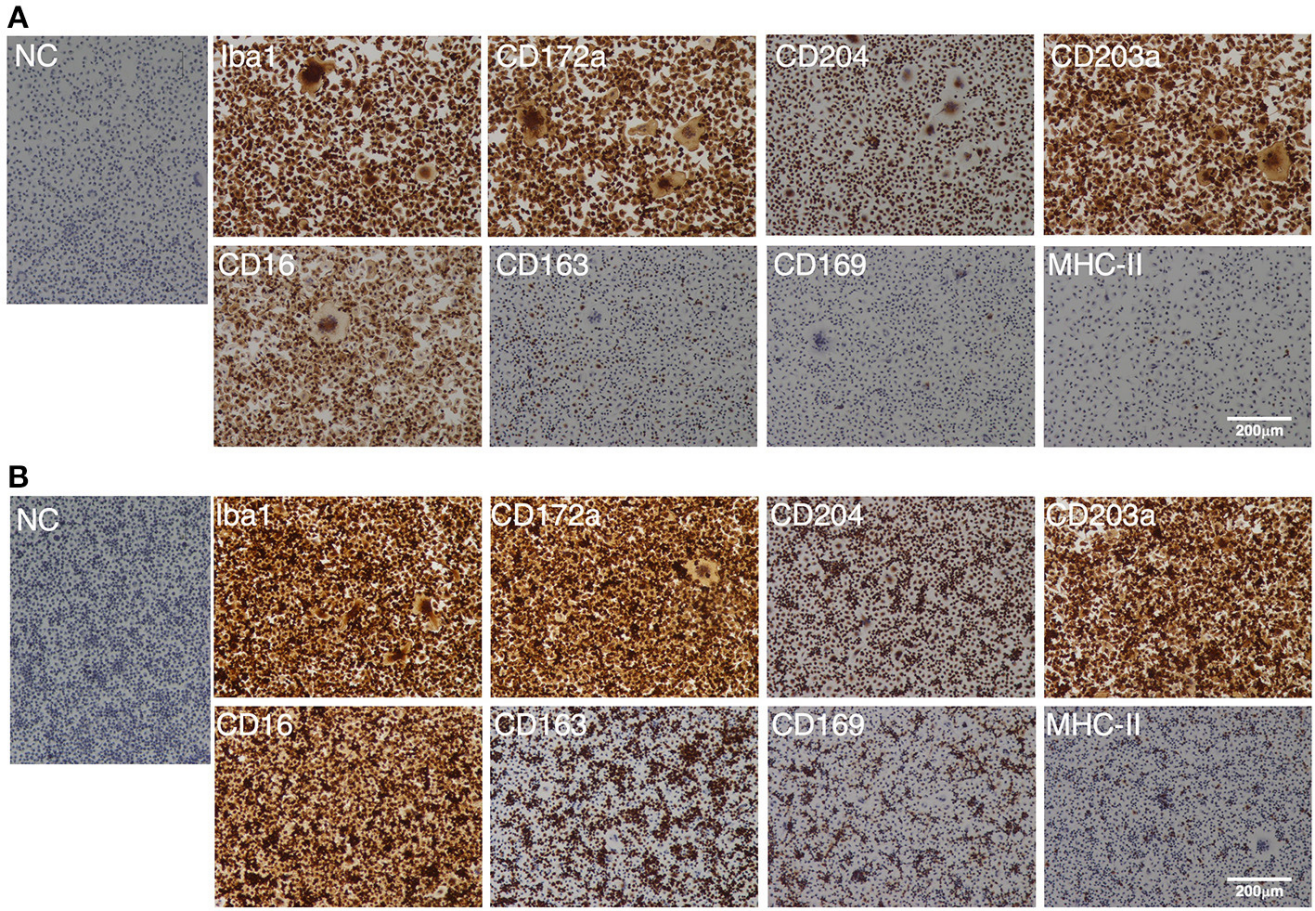
Immunocytochemical characterization of IPIM. The IPIM were seeded in 8-well-chamber slides and cultured for 1 day **(A)** or 3 days **(B)**. Then, the cells were fixed using 4% paraformaldehyde phosphate buffer solution, and immunostained with specific antibodies against cell markers of macrophages (Iba1, CD172a, CD204, CD203a, and CD16) or specific subpopulations of macrophages (CD163, CD169, and MHC-II) (*brown*). No specific staining was observed if the cells were treated without primary antibodies [NC: negative control in **(A,B)**]. All nuclei were counterstained with hematoxylin (*blue*). Images are representative of three independent experiments.

As with the PIM, the IPIM were clearly positive for macrophage markers (Iba-1, CD172a, and CD204) (Figure 3). They were also positive for other macrophage cell surface markers, CD203a and CD16 (Figure 3). Some populations of IPIM were positive for markers of specific subpopulations of macrophages (CD163, CD169, and MHC-II) (Figure 3).

### Inflammatory response of IPIM

**Figure 4:**
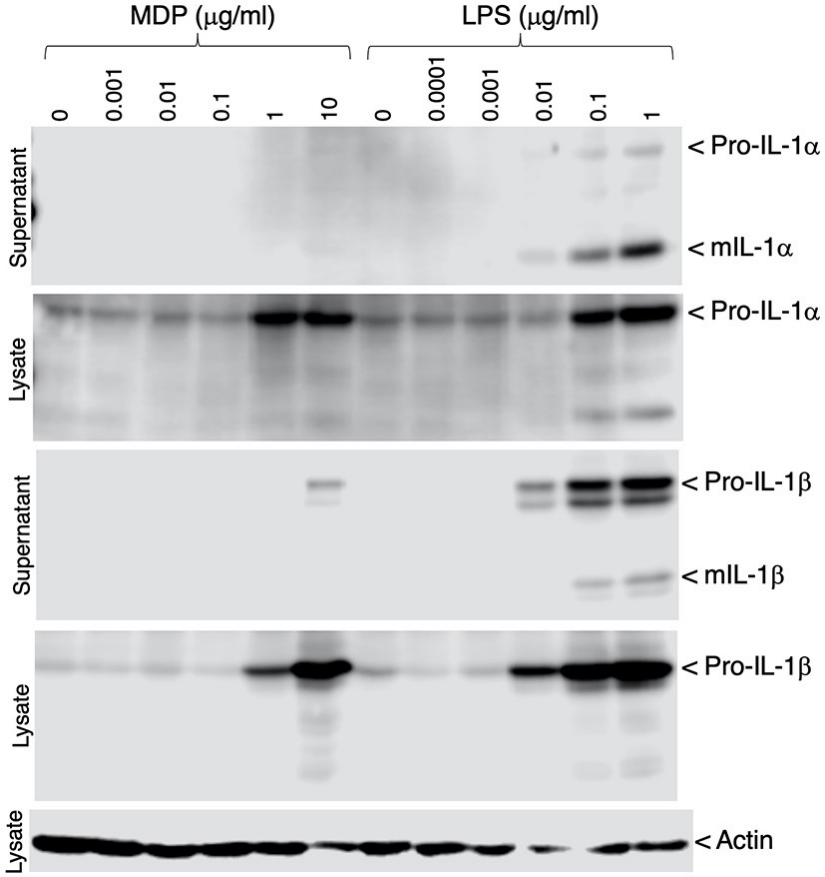
Production of IL-1α and IL-1β in MDP- or LPS-stimulated IPIM. Dose-dependent production of pro-IL-1α and pro-IL-1β was detected in cell lysates from IPIM that had been stimulated with MDP or LPS for 3 days (*second and fourth panels*). The secretion of their mature forms (mIL-1α and mIL-1β) into the culture supernatant was detected in the LPS-treated IPIM (*first and third panels*). Actin was detected as an internal control for protein loading in each lane (*fifth panel*). Data are representative of three independent experiments.

To verify that inflammatory responses could be induced in the IPIM, the effects of bacterial cell wall components, muramyl dipeptide (MDP) and lipopolysaccharide (LPS), were investigated. These stimuli induced the production of the precursor form of IL-1α and IL-1β (pro-IL-1α and pro-IL-1β), potent pro-inflammatory cytokines, in a dose-dependent manner (Figure 4, second and fourth panels). In particular, LPS triggered the secretion of their mature active forms (mIL-1α and mIL-1β) into the culture supernatant (Figure 4, first and third panels).

### Susceptibility of IPIM to ASFV infection

**Figure 5:**
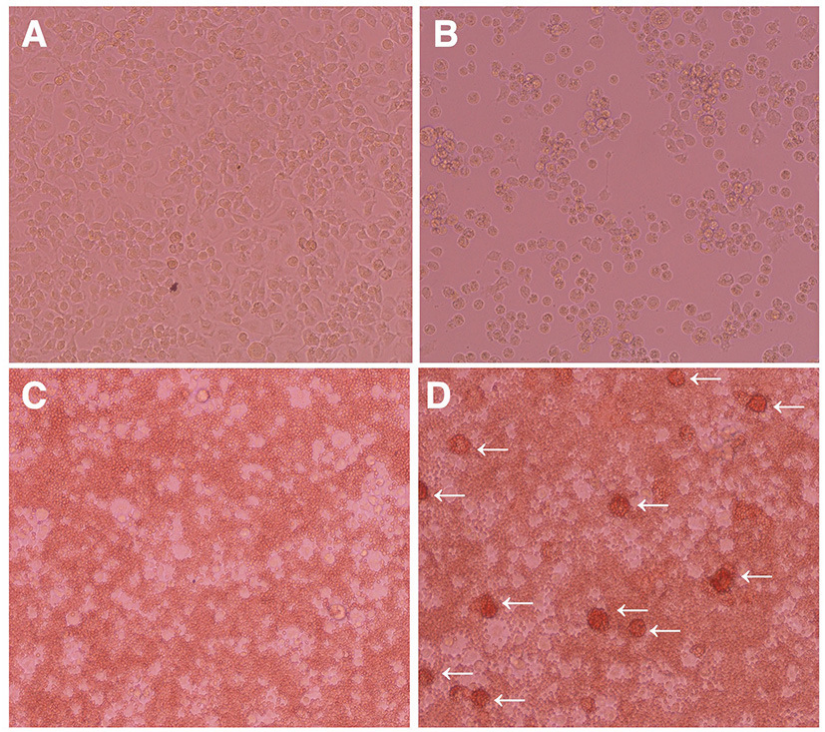
Cytopathic effects and hemadsorption assays of ASFV-inoculated IPIM. Cells were mock-inoculated **(A,C)** or inoculated with the Armenia07 isolate (MOI = 0.1) **(B,D)** in the absence **(A,B)** or presence **(C,D)** of porcine red blood cells. Cytopathic effects **(B)** and rosette formation [**(D)**, white *arrows*] were detected in Armenia07-infected cells at 2 dpi. Images are representative of at least three independent experiments.

We investigated whether IPIM are susceptible to ASFV infection. When the IPIM were inoculated with a virulent ASFV field isolate, Armenia07, CPE were clearly observed at 2 dpi (Figure 5B) compared with the findings obtained for mock-infected cells (Figure 5A). Rosette formation was also observed in Armenia07-inoculated IPIM (Figure 5D), but not in mock-infected cells (Figure 5C), in a HAD assay.

**Figure 6:**
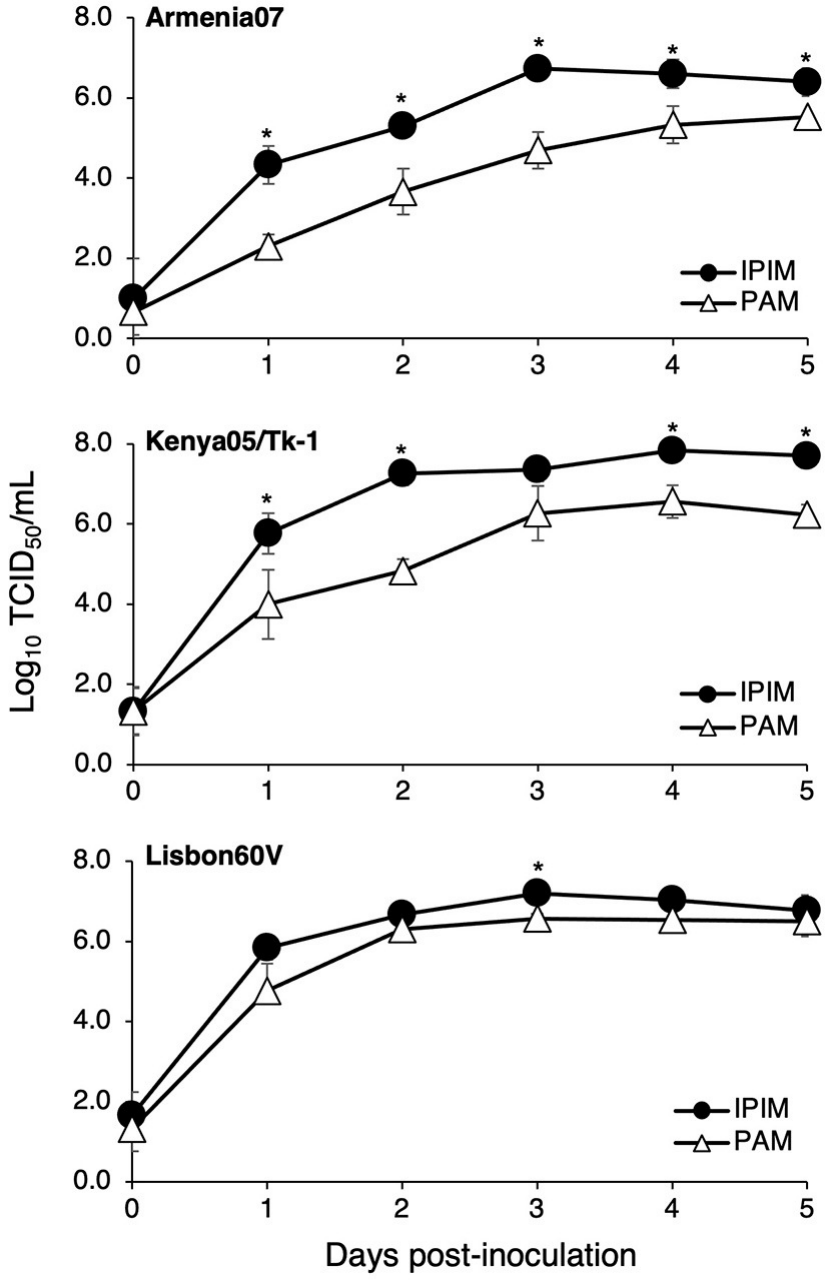
Comparison of the ASFV production in IPIM and PAM. Cell cultures were infected with Armina07 (*upper*), Kenya05/Tk-1 (*middle)*, and Lisbon60V isolates (*lower*) (MOI = 0.01). The culture supernatant samples were collected at the indicated timepoints. Viral production in the IPIM (*closed circle*) and PAM (*open triangle*) cell cultures were estimated by titration experiments with the IPIM. Data represent the mean and standard deviations of three experiments. Asterisks indicate statistically significant differences in the viral production between the IPIM and PAM cell cultures (*p < 0.05).

Furthermore, ASFV titration was performed in IPIM cell cultures using the CPE assay. It is noteworthy that the ASFV field isolates Armenia07, Kenya05/Tk-1, and Espana75 were propagated in IPIM cell cultures and reached maximal titers of 10^6.6^, 10^7.2^, and 10^6.3^ TCID_50_/mL (the 50% tissue culture infectious dose per mL) at 7 dpi, respectively. A Vero cell-adapted ASFV isolate, Lisbon60V, showed the highest maximal titer of 10^8.4^ TCID_50_/mL among the ASFV isolates tested. Similar results were obtained when the virus titration was evaluated using the HAD assay; i.e., the Armenia07, Kenya05/Tk-1, Espana75, and Lisbon60V isolates exhibited maximal titers of 10^6.5^, 10^7.2^, 10^6.5^, and 10^8.3^ HAD_50_/mL (HAD units yielding a 50% of cumulative infection per mL) at 7 dpi, respectively. ASFV titration was also performed in PAM cell cultures using the HAD assay. As a result, the maximal titers of the Armenia07, Kenya05/Tk-1, Espana75, and Lisbon60V ASFV isolates were 10^6.8^, 10^6.4^, 10^6.2^, and 10^6.6^ HAD_50_/mL at 7 dpi, respectively. A virus growth assay showed that Armenia07, Kenya05/Tk-1, and Lisbon60V ASFV isolates replicate well in IPIM cell cultures in a time-dependent manner, that are equivalent to or more than their replication in PAM cell cultures (Figure 6).

### Susceptibility of IPIM to PRRSV infection

**Figure 7:**
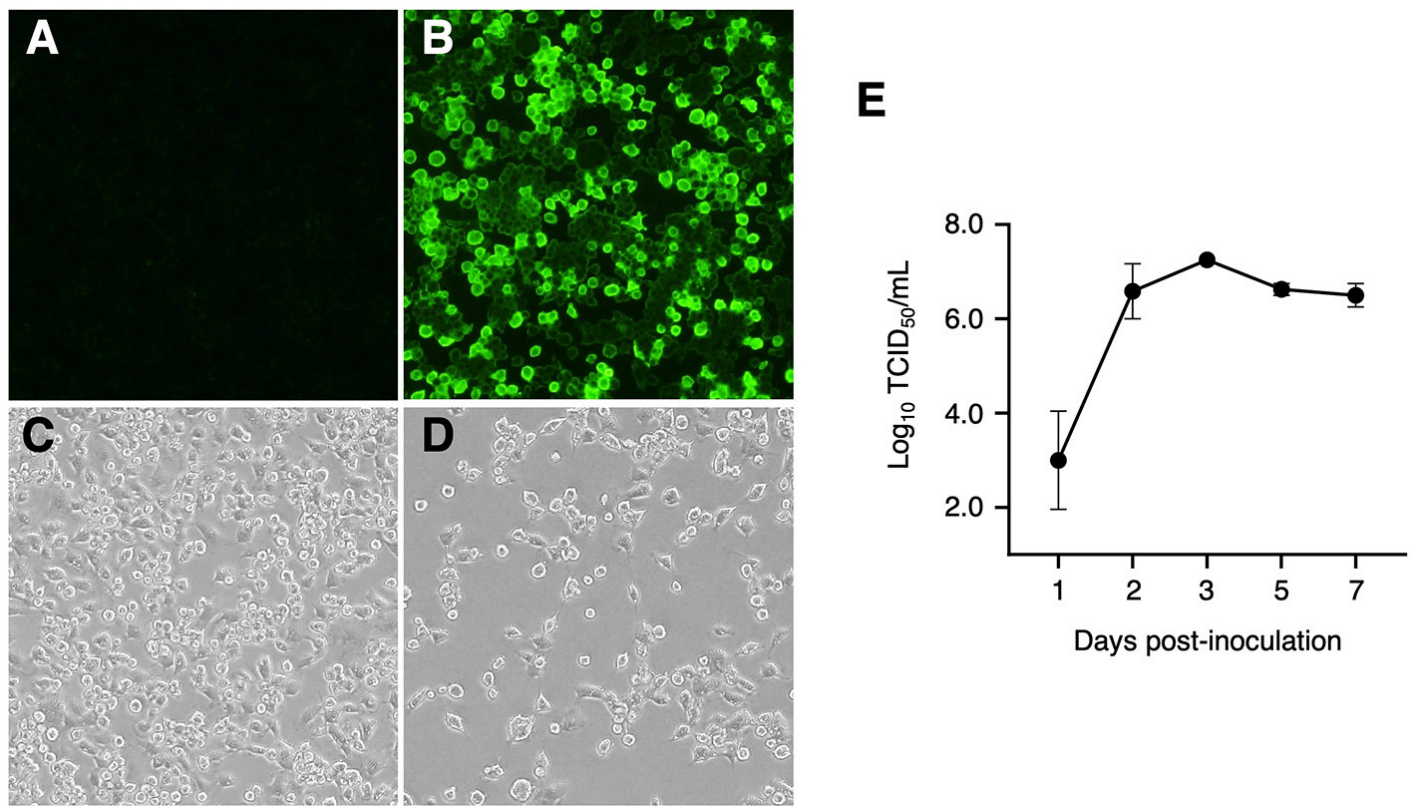
Detection of PRRSV antigens using an immunofluorescence assay, cytopathic effects, and viral growth curve for PRRSV-inoculated IPIM. A direct immunofluorescence assay was performed with FITC-conjugated anti-PRRSV monoclonal antibodies using mock-inoculated **(A)** and PRRSV-inoculated **(B)** cells at 3 dpi. The cytopathic effects of the PRRSV were also evaluated in mock-inoculated **(C)** and PRRSV-inoculated **(D)** cells at 7 dpi under a phase-contrast microscope. After inoculation with PRRSV, culture supernatant samples were collected at the indicated timepoints and used for titration experiments with the IPIM **(E)**. Data are expressed as the mean ± SEM of three experiments **(E)**.

We also investigated whether IPIM are susceptible to PRRSV infection. When IPIM were inoculated with the EDRD-1 strain of the PRRSV, PRRSV antigen was detected in the cytoplasm from 1 dpi and peaked at 3 dpi (Figures 7A,B). CPE were visible from 3 dpi and became evident at 5-7 dpi (Figures 7C,D). A virus growth assay demonstrated that the PRRSV EDRD-1 strain replicates well in IPIM cell cultures, with maximal titers of around 10^7^ TCID_50_/mL seen at 3 dpi and maintained the high titers until 7 dpi (Figure 7E).

## Discussion

Most previous methods for intestinal macrophage isolation require sophisticated skills and equipment, such as a cell sorter and a centrifugal elutriator (25, 26). In addition, the isolated macrophages do not proliferate in primary culture, and thus need to be prepared at time of use for every experiment. In this context, our method is beneficial that PIM are isolated by the general primary cell culture technique alone, and their propagation is elicited to some extent under mixed culture conditions, and their cultures are maintained for a few months until use. More quantitative analyses are required to determine the proliferative capacity of PIM under mixed primary culture conditions. Also, we realized that the usage of fetal porcine intestinal tissue easily minimizes the risk of contamination of the primary cell culture by intestinal bacteria.

Although the total number of PIM collected by the presented protocol was limited, the yields were sufficient to conduct several macrophage characteristic analyses, and establish their immortalized cell line. More amounts of intestinal tissues need to be treated to obtain an increased number of PIM. Since other types of cells, such as epithelial/mesenchymal cells and lymphocytes including B cells known as professional antigen-presenting cells (APC) do not attach to the NTC-dishes, they are readily washed away after medium changes when intestinal mixed cells were seeded in NTC-dishes. As another professional APC, dendritic cells (DC) are monocyte/macrophage-lineage cells, and thus, may express some macrophage markers, such as Iba1, CD172a and CD204. Since the intestine contains the largest pool of macrophages in the body (6), it is expected that PIM shown in this study mainly consist of macrophage populations rather than DC. To strictly identify intestinal macrophages or DC, thorough multi-parameter analysis with several surface markers needing to be examined in combination seems to be required (27).

Regarding the origin of PIM, a previous study suggested that most tissue-resident macrophages are derived from primitive macrophages found within the yolk sac or fetal liver (28). The intestinal macrophage population seems to be established with the primitive macrophages prior to birth and is continuously replaced by BM-derived macrophages during adulthood (8). As it involves the use of porcine fetal intestine tissue, it is suggested that PIM collected using the present protocol retain the features of primitive intestinal macrophages.

In addition, we established a novel immortalized IPIM cell line, which retains various features of primary macrophages, like the IPKM cell line (18). Immunostaining suggests that some populations of IPIM expressed marker proteins for specific subpopulations of macrophages, such as CD163, CD169 and MHC-II (29). CD163 is mainly expressed in macrophages and used as a phenotypic marker of anti-inflammatory M2 subtypes (30), implying that IPIM contain M2 macrophages. IPIM also contain CD169-positive macrophages that are a unique type of macrophage subset that differ from pro-inflammatory M1 and anti-inflammatory M2 macrophages (31). The finding that few populations of IPIM express MHC-II, a marker of mature DC, supports the notion that IPIM retain the features of macrophages rather than those of DC.

To date, several porcine macrophage cell lines have been developed for *in vitro* immunological and virological studies, since macrophages are the main target cells of various porcine viruses, including ASFV and PRRSV. For instance, three cell lines of immortalized PAM (IPAM) were established by transferring a SV40LT gene to primary PAM *via* transfection with the pSV3neo plasmid (32). Porcine blood monocyte-derived macrophage cell lines were also developed without gene transfer (33, 34). In addition, Zuckermann macrophage (ZMAC) cells are a spontaneously occurring monocytic cell line derived from the bronchoalveolar lavage of porcine fetal lungs, whose expansion is supported by supplementation with recombinant murine macrophage colony-stimulating factor (35, 36). In comparison with these previously established cell lines, IPIM are unique in terms of their intestinal macrophage origin, and they could be used in combination with porcine small intestine-derived epithelial and/or stromal cells to develop *in vitro* models that mimic the intestinal microenvironments of swine.

ASFV is a highly pathogenic virus with a marked tropism for cells of the monocyte-macrophage lineage (37). However, it was reported that the level of infection and production of ASFV isolates were much lower in IPAM than in primary PAM (38), suggesting that the induction of immortalization may reduce the ability of ASFV to infect macrophages. Until now, the lack of reliable porcine cell lines that are susceptible to ASFV infection has limited research on ASFV-host interactions and the development of live-attenuated vaccine (LAV) strains. To overcome this, recent studies have proposed promising porcine macrophage cell line candidates, which may be useful for *in vitro* studies of ASFV. Portugal et al. reported that ZMAC cells are susceptible to infection by field isolates of ASFV, and titrations in these cells reached similar levels to those seen in porcine BM-derived primary macrophages for all of the isolates tested (35). Our recent study also demonstrated that IPKM can support the replication of ASFV field isolates, and the growth of these isolates occurred faster in the IPKM cell culture than in the PAM cell culture (22). Here, we additionally showed that the IPIM cell line is highly susceptible to infection by ASFV field and Vero cell-adapted isolates. Further experiments will be required to explore the most suitable usage for each cell line in ASFV research, e.g., virus isolation and propagation, diagnostic use, or LAV development and production.

PRRSV is a globally ubiquitous porcine viral pathogen that causes major economic losses to the swine industry worldwide (39). Simian kidney-derived MARC-145 cells are frequently used to isolate and propagate PRRSV (40) and to develop its LAV strains (41). However, since PRRSV exhibits highly restricted tropism for cells of the monocyte-macrophage lineage, primary PAM are generally believed to be superior to MARC-145 cells for the isolation of PRRSV field strains. In this context, porcine macrophage cell lines that are capable of supporting the replication of different PRRSV strains have been developed. ZMAC cells can be used for the production of an LAV strain of PRRSV (36) and allow better isolation of a wide range of PRRSV field strains (42). IPKM are susceptible to infection by genetically diverse Japanese field strains and commercial LAV strains of PRRSV type 2 (43). Both ZMAC cells and IPKM are reported to be more useful for isolating PRRSV from field samples and diagnostic applications than MARC-145 cells (42, 43). Since IPIM were also shown to be susceptible to infection by a PRRSV field strain and exhibited a high infection titer (10^7^ TCID_50_/mL), this cell line is expected to be useful for isolating PRRSV from field samples and developing new LAV strains. Furthermore, it is reported that PRRSV infection impairs intestinal integrity by damaging physical and immune barriers in the intestine by inducing inflammation, which may be related to the regulation of the gut-lung axis (44). Thus, it is conceivable that IPIM or PIM are valuable tools for investigating the mechanisms by which PRRSV affects intestinal mucosal inflammation and the pathogenesis of PRRSV-induced diarrhea.

IPIM exhibited pro-inflammatory reactions in response to MDP and LPS. In particular, LPS triggered the secretion of mature active forms of IL-1α and IL-1β into the culture supernatant, suggesting the expression of functional porcine inflammasome system (45). It is also speculated that ASFV or PRRSV infection induces the activation of IPIM, and affects their inflammatory responses. Comprehensive expression analysis such as RNA-sequencing would be helpful for identifying the inflammatory genes affected by these virus infections in the IPIM.

In conclusion, primary PIM and the IPIM described here represent practical and reliable *in vitro* tools for elucidating the innate immune functions of macrophages and cellular communication between intestinal epithelial cells and innate immune cells in swine. Furthermore, these macrophages may be useful for investigating the host-pathogen interactions that occur in porcine diarrhea epidemics. IPIM are also expected to be a valuable tool for the development and production of LAV strains, which can help prevent porcine viral diseases.

## Data availability statement

The original contributions presented in the study are included in the article/supplementary material, further inquiries can be directed to the corresponding authors.

## Ethics statement

The animal study was reviewed and approved by the animal care committee of the Institute of Agrobiological Sciences (#H28-P04) and the National Institute of Animal Health (NIAH) (#20-046), National Agriculture and Food Research Organization (NARO).

## Author contributions

TT, KM, and AM conceived and designed the experiments, performed the experiments, analyzed the data, and wrote the manuscript. TT, KM, AM, SS, MT, TK, and HU contributed reagents, materials, and analysis tools. All authors reviewed the manuscript, contributed to the article, and approved the submitted version.

## Funding

This study was supported by a grant from a project of the NARO Bio-oriented Technology Research Advancement Institution (Research Program on the Development of Innovative Technology, No. 01002A).

## Conflict of interest

The authors declare that the research was conducted in the absence of any commercial or financial relationships that could be construed as a potential conflict of interest.

## Publisher's note

All claims expressed in this article are solely those of the authors and do not necessarily represent those of their affiliated organizations, or those of the publisher, the editors and the reviewers. Any product that may be evaluated in this article, or claim that may be made by its manufacturer, is not guaranteed or endorsed by the publisher.
